# Free-flight and tracking experiments of a multi-parabola laser propulsion vehicle

**DOI:** 10.1038/s41598-025-00429-0

**Published:** 2025-05-03

**Authors:** Masayuki Takahashi, Yuya Hayadate, Koichi Mori, Akihiro Hayakawa

**Affiliations:** 1 Department of Aerospace Engineering, Tohoku University, Sendai, 980-8579 Japan; 2 Department of Aerospace and Marine Engineering, Osaka Metropolitan University, Sakai, 599-8531 Japan; 3 Institute of Fluid Science, Tohoku University, Sendai, 980-8577 Japan

**Keywords:** Laser propulsion, Free flight, Tracking system, Aerospace engineering, Electrical and electronic engineering, Mechanical engineering

## Abstract

A free-flight experiment involving a multi-parabola laser propulsion vehicle was conducted using repetitive laser pulses at a repetition frequency of 50 Hz and an energy of 4.93 J per pulse. Observations made with a camera indicated that the vehicle achieved a maximum altitude of 110 mm; however, it deviated from the laser beam line due to initial misalignment of the laser setup. To improve flight stability, we developed a tracking system to monitor the vehicle’s motion and control the laser irradiation position. Performance requirements were assessed based on the free-flight experiment data, revealing that the vehicle attained a maximum translational speed of 0.08 m/s before deviation occurred. Our tracking system was evaluated to have a trackable speed of 0.09 m/s, meeting the performance requirements for free flight and capable of stabilizing the repetitive pulse flight of a laser propulsion vehicle.

## Introduction

The demand for rocket launches of small satellites is dramatically increasing due to various types of space missions proposed for university research, national security, and economic activities. However, the launch cost of rockets remains high because conventional rockets rely on chemical combustion engines, which have low reusability. Additionally, a significant amount of fuel is required to sustain the combustion reaction and high thrust in a chemical engine, further increasing launch costs. To address these challenges and significantly reduce launch costs, beamed-energy propulsion technology has been proposed as a novel transportation system for outer space. In this concept, a high-average-power repetitive pulse beam is emitted from a ground-based station to a vehicle flying at high altitude. Upon irradiation, the beam is focused by a parabolic mirror mounted on the vehicle, creating a high electric-field region and inducing gas breakdown through an electron avalanche process. This process generates a strong blast wave as the energy of the laser-induced plasma is transferred to neutral particles. The vehicle gains thrust by receiving the high pressure of the blast wave without using a gas-burning engine or fuel for combustion. A rocket launch using beamed-energy propulsion is then achieved by repeating the pulse beam irradiation, gas breakdown, and shock wave generation processes. The fundamental difference between chemical rockets and beamed-energy propulsions is that chemical rocket engines are internal combustion engines, whereas beamed-energy propulsions are external combustion engines. Additionally, another difference is that the fuel can be completely removed. Therefore, the thrust-to-mass ratio and specific impulse limitations of conventional engine systems can be avoided in beamed-energy propulsion.

In this launch concept, microwave rockets and laser propulsion systems are proposed, which can be differentiated based on the frequency of the incident beam. This study focuses on the laser propulsion system, but before delving into its specifics, we will outline the advantages and disadvantages of each concept. In the microwave rocket concept, an intense millimeter-wave pulse beam with a longer pulse width, longer wavelength, and lower energy fluence than a laser beam irradiates the vehicle to induce plasma and shock waves^1–25^. Due to the longer pulse width of the millimeter-wave beam, a quasi-steady detonation-like shock wave structure is formed inside the rocket nozzle during the beam irradiation interval, instead of generating a blast wave. This detonation-like shock wave, when received by a longer nozzle, enables the microwave rocket to achieve a momentum coupling coefficient comparable to that of laser propulsion. In addition to the thruster design difference, microwave plasma has stronger non-equilibrium dynamics than of laser-induced plasma, which is of significant research interest^23–25^. The advantage of a microwave rocket lies in the lower construction cost of its beam oscillator compared to a laser beam oscillator^3^. However, the disadvantage is beam divergence during transmission to high altitudes, as the divergence angle of the beam increases with wavelength, as follows; $w(h)=\sqrt{1+\biggl (\frac{\lambda h}{\pi w_0^2}\biggr )^2}$, where *w* is the beam waist, $\lambda$ is the beam wavelength, *h* is the propagation distance, and $w_0$ is the minimal beam waist. Due to this large divergence angle, using millimeter-wave irradiation as the energy source requires a larger beam receiver diameter than that for laser propulsion, increasing the rocket’s weight and decreasing payload efficiency. Conversely, the short wavelength of the laser pulse in the laser propulsion system is advantageous because it minimizes beam divergence during long-distance transmission, thereby reducing the vehicle’s weight. To utilize the advantages of remote energy transfer with low beam divergence, this study focuses on laser propulsion rather than microwave rockets^26–42^. In future launch missions, microwave rockets could be combined with laser propulsion to benefit from the advantages of each. Rockets will be driven by microwave rockets when flying at low altitudes and switched to laser propulsions as higher altitudes; these prospects will be discussed in a forthcoming paper.

There are various research topics in laser propulsion systems, such as the enhancement of axial thrust and the physics of laser-induced plasma, as indicated in the previous works^35–42^. However, this study focuses on flight stability, specifically the “beam-riding” dynamics that keep the vehicle aligned with the beam axis when an offset between the laser and vehicle axes occurs due to disturbances. As the previous studies for the beam-riding flight of the laser propulsion, the demonstration of the “lightcraft” laser propulsion vehicle was the most successful experiment, which achieved a flight altitude of 70 m^26–28^. As the other concept for the beam-riding flight, a bell-shaped thruster was also proposed by DLR, and the flight experiments were conducted by the repetitive pulse laser^29–31^. In beam-riding flight dynamics, the lightcraft-type vehicle employs a ring-shaped cowl to generate a re-centering force, feeding the vehicle’s position back to the center of the beam when the incident laser beam has a lateral offset relative to the vehicle axis. Despite this, flight experiments indicated that deviations from the laser beam line occurred during free flight in the air^26–28^.

The flight simulator for the lightcraft was developed to reproduce and understand fundamental behavior of the beam-riding flight, as indicated in the previous papers^32–34^. Additionally, we revealed the detailed deviation mechanism of the lightcraft through a six degree-of-freedom (6-DOF) flight simulation^43–45^, showing that a re-centering force was obtained and stable flight was maintained when the angular offset between the incident laser and vehicle axes was small. However, as the angular offset increased during free flight, the re-centering force diminished, causing the lightcraft to deviate from the laser axis. Specifically, our simulation^43^ shows that the angular offset of approximately 3.0 degrees can be a criteria for the stable beam-riding flight, because the centering feedback force was significantly decreased and deviation from the laser beam line occurred when the angular offset of the flying vehicle exceeded this criterion. Therefore, suppressing the angular offset is crucial for maintaining beam-riding flight stability. Although redesigning the vehicle shape can enhance the angular restoring force, an alternative approach involves active control through flight monitoring and feedback. In our previous studies^43–45^, an active laser control method was proposed, which uses a genetic algorithm to find the optimal laser irradiation position and adjusts it in synchronization with the vehicle’s position and posture, thereby suppressing angular offset during free flight. The 6-DOF flight simulation with this active control method demonstrated that continuous thrust generation and stable flight were achieved by maintaining a small angular offset. However, the proposed active control system for beam-riding flight was only evaluated through simulation, and experimental development and performance evaluation of the active control system have not yet been conducted.

In this study, we developed an active control system and experimentally verified the tracking performance of a moving laser propulsion vehicle. Before evaluating the tracking system’s performance, we conducted a free-flight experiment with a laser propulsion vehicle using repetitive pulses to determine the performance requirements for the tracking system, including the allowable response time for vehicle position detection and the speed for laser position actuation. The free-flight experiment was conducted using the “multi-parabola thruster” proposed by us^46^, rather than the lightcraft (a detailed discussion of the multi-parabola thruster’s beam-riding performance will be presented in a forthcoming paper). The multi-parabola thruster is shaped as a combination of the lightcraft with a cowl and the bell-shaped thruster proposed by DLR^29–31^. The lateral feedback force can be obtained by interactions between the ring-shaped blast wave and cowl. The main thrust and angular restoring impulses can be generated by a simple parabolic mirror such as a bell-shaped nozzle. We have previously conducted the impulse measurement experiment for the multi-parabola thruster using a single pulse laser, which indicated that the lateral and angular feedback impulses could be obtained by blast waves^46^. Following the free-flight experiment, we evaluated the tracking system’s performance, which consists of a stereo camera and a mirror actuator, by attaching the multi-parabola thruster to a robotic arm to simulate translational motion. This tracking experiment assessed whether the system met the performance requirements for maintaining beam-riding flight.

## Results in free-flight experiment

### Confirmation of initial laser offset and nonparallel beam formation in our optical setting

Ideally, no laser offset would occur in the optical setting. However, in practice, initial laser offsets can result from alignment errors. In our setup, initial laser offsets were introduced in both lateral and angular directions. Additionally, a parallel beam was not achieved in this experiment, leading to beam focusing during vertical upward propagation. Consequently, laser plasma was induced at an altitude of 135 mm, even when the laser was irradiated into empty space. Therefore, beam-riding flight beyond 135 mm could not be achieved, as the laser energy did not propagate beyond this altitude. This section discusses the initial laser offset and focusing, with free-flight dynamics limited to an altitude of 135 mm.

**Fig. 1:**
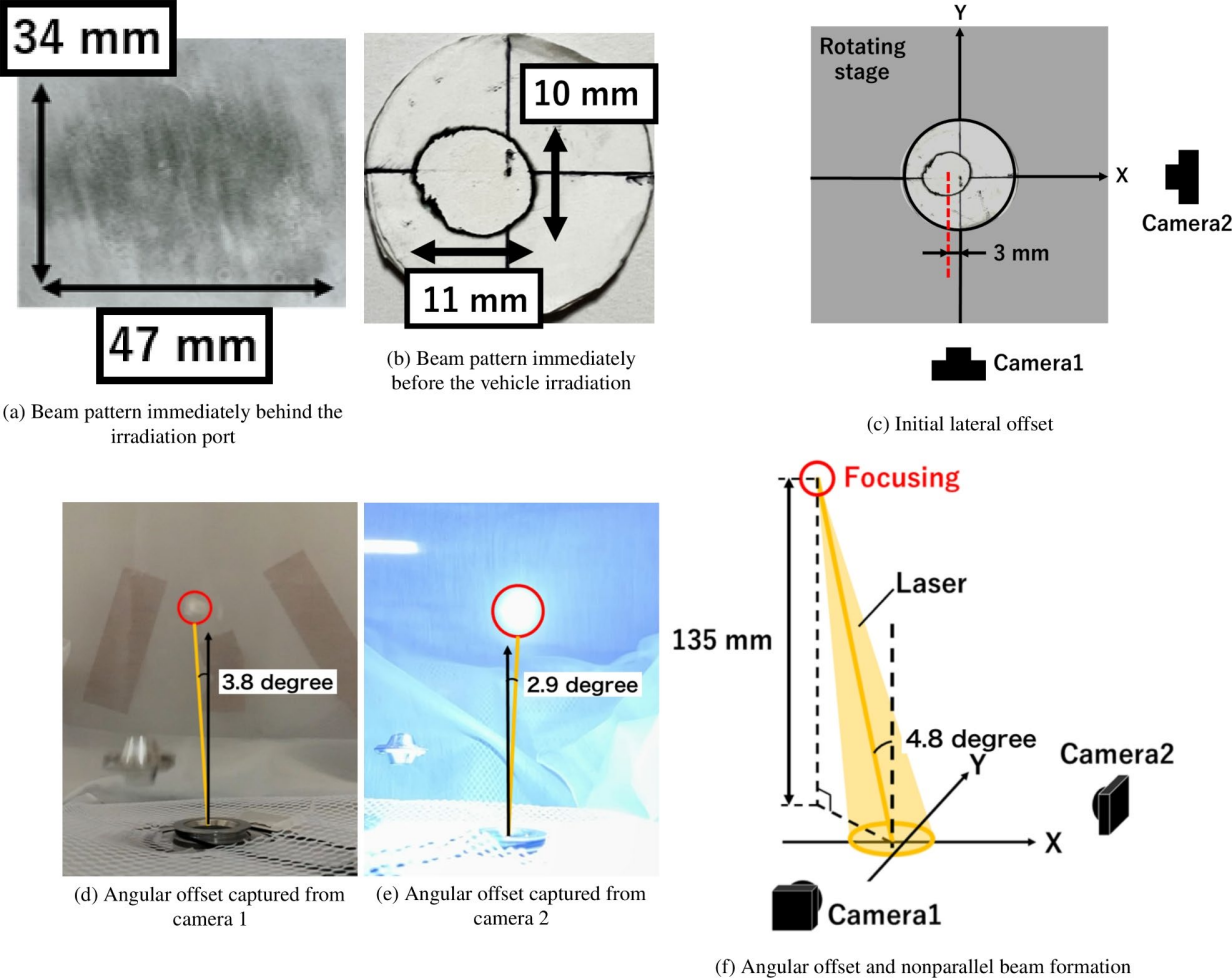
Beam pattern and initial offsets.

Figure 1a,b shows the beam patterns on the burn paper, captured just behind the irradiation port and just before the beam was directed onto the vehicle. In the beam pattern image shown in Fig. 1c, the *X*- and *Y*-axes represent the depths observed by cameras 1 and 2, respectively, with the vehicle center set at the origin of the *XY*-plane. The *Z*-axis extends vertically from the origin, corresponding to the flight direction. The beam center was shifted by 3 mm in the $-X$-direction due to misalignment, which was considered the initial lateral offset. Additionally, the initial angular offset and nonparallel beam irradiation were confirmed by observing air breakdown without the vehicle. Figure 1d,e show an air-breakdown spot after the vehicle deviated from the laser beam line, indicating that laser breakdown occurred even when the laser did not irradiate the vehicle. This implies that a parallel beam was not formed, and laser focusing occurred due to the optical device settings. Furthermore, the breakdown spot was not on the *Z*-axis (indicated by the black arrow in Fig. 1d,e), confirming the existence of an initial angular offset. The angular offset measured 3.8 degrees and 2.9 degrees from cameras 1 and 2, respectively, with the angle between the laser and *Z*-axes being 4.8 degrees (Fig. 1f). In summary, there was a lateral offset of 3 mm in the $-X$-direction and an angular offset of 4.8 degrees. The beam was focused at a flight altitude of 135 mm, and the vehicle flight dynamics under these laser conditions were analyzed. To achieve higher flight altitudes, it is essential to avoid initial offsets and ensure a parallel beam, which will be addressed in a forthcoming paper.

### Flight dynamics under repetitive pulses

**Fig. 2:**
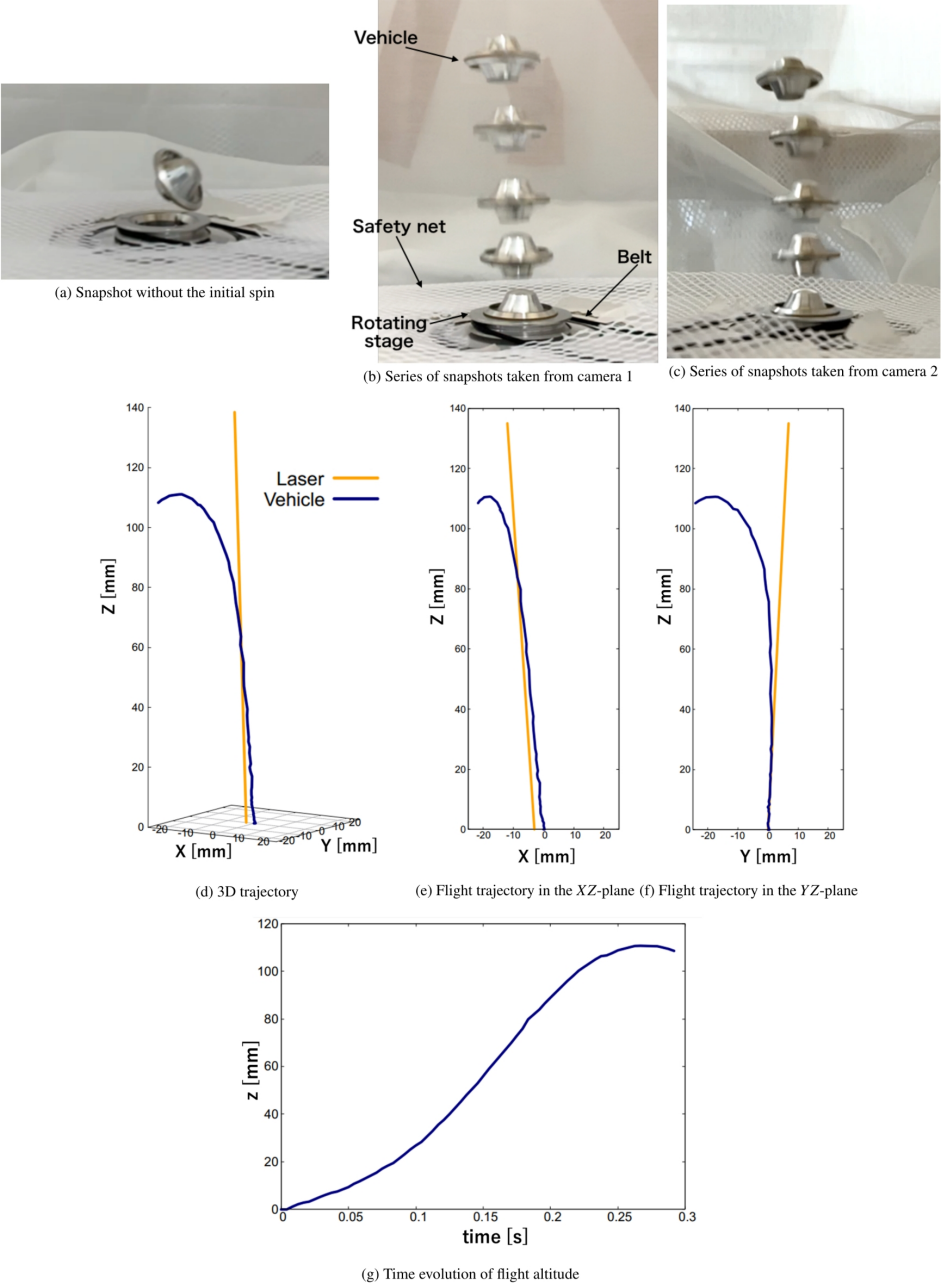
Series of snapshots of the free-flight vehicle and trajectory.

The flight dynamics were evaluated by placing the vehicle on the rotating stage. Ideally, stable flight should be achieved without initial spin; however, without the initial spin, stable beam-riding flight was not attained. The angular offset of the vehicle rapidly increased when the first pulse was applied as shown in Fig. 2a. This increase was due to the initial lateral offset of the laser irradiation. Additionally, the vehicle’s small moment of inertia, resulting from its reduced weight, amplified the effect of the initial laser offset, causing a rapid increase in the angular offset immediately after the first pulse. The feedback from this large change in the vehicle altitude is difficult, as predicted in our previous 6-DOF simulations^43–45^, resulting in unsustainable flight.

In contrast, an initial spin rate was gradually increased, showing that stable beam-riding flight over several pulses was achieved when an initial spin of 7,800 rpm was applied to the vehicle. To clarify the criteria for the active control system, the flight trajectory and velocity were examined under this initial spin condition. Figure 2b,c show a series of images of the free-flight vehicle captured by cameras 1 and 2, respectively. Based on these snapshots, a three-dimensional (3D) flight trajectory was evaluated, as shown in Fig. 2d–f. The time evolution of the flight altitude is illustrated in Fig. 2g, indicating that a maximum flight altitude of 110 mm was reached.

We now consider the detailed flight dynamics of the multi-parabola thruster based on the trajectory data in Figs. 2e,f. The trajectories in the *XZ*- and *YZ*-planes (Fig. 2e,f) show that the vehicle moved in the $-X$-direction and followed the laser axis (yellow line) within an altitude range of 0–40 mm, due to the centering feedback force resulting from the lateral offset in the $-X$-direction. This centering feedback force was generated because the side mirror created a ring-shaped blast wave with non-uniform energy density, pushing the cowl as per the original design concept of the multi-parabola thruster. However, the deviation from the laser beam line began in the altitude range of 40–100 mm. The vehicle completely deviated from the laser axis at an altitude of 100 mm and crashed after inertial forces caused it to climb to an altitude of 110 mm.

**Fig. 3:**
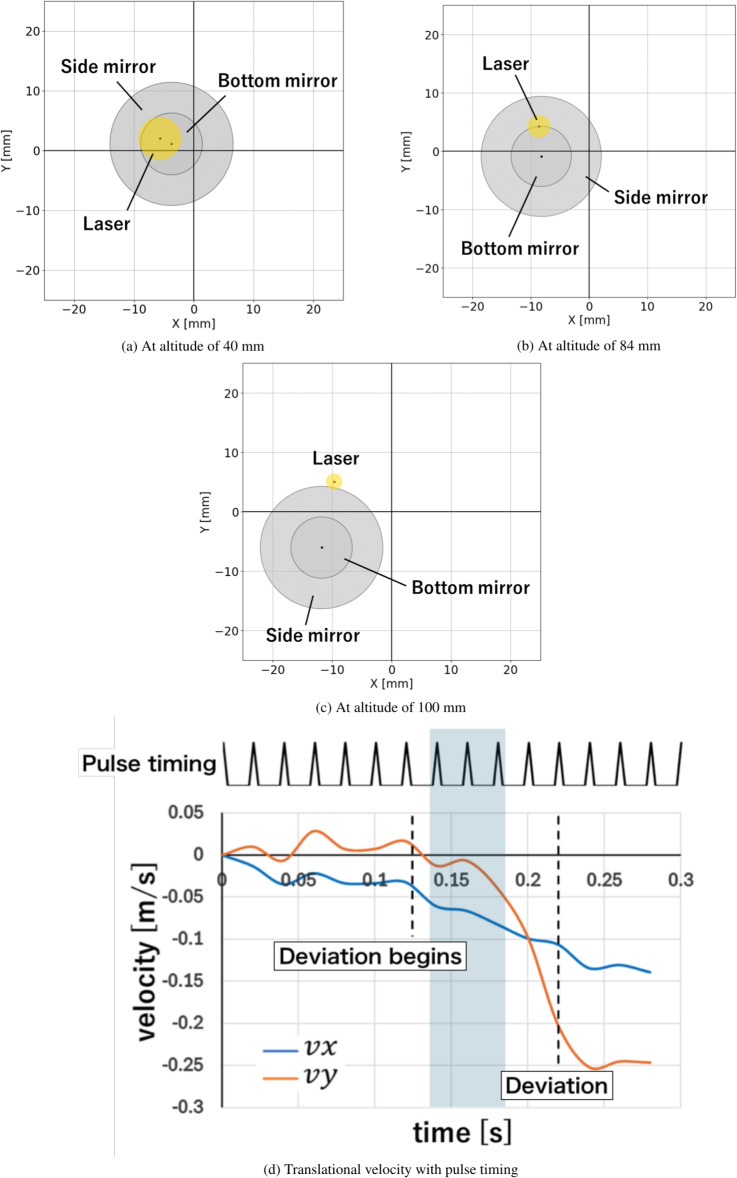
Relationship between the vehicle and laser axis depending on flight altitude. Translational velocity with pulse timing.

This deviation process from the laser beam line can be understood by examining the detailed relationship between the vehicle and the laser beam, as illustrated in Fig. 3. At altitudes below 40 mm, a centering force was generated because the laser beam was irradiated onto the side mirror, reducing the lateral offset between the vehicle and beam centers. This lateral feedback behavior due to the cowl was similar to that of the lightcraft, as predicted in our previous simulations^43–45^. However, at an altitude of 40 mm, the laser-irradiation position shifted from the side to the center mirrors (Fig. 3a). In addition to this position shift, as shown in Fig. 3b, the beam diameter irradiated onto the vehicle decreased as the flight altitude increased from 40 to 84 mm, due to the lack of a parallel beam in our optical setup, causing beam focusing towards an altitude of 135 mm. Consequently, the centering feedback force was not generated at altitudes between 40 and 84 mm because the reduced beam diameter prevented irradiation onto the side mirror. Additionally, the initial angular offset of 4.8 degrees was not compensated by the gyro rotation around the vehicle axis. This angular offset caused a force that shifted the vehicle position in the *XY*-plane away from the beam center, due to a deformed blast wave generated inside the main parabolic mirror^46^, pushing the vehicle in both the *X*- and *Y*-directions. As a result, the lateral offset between the vehicle and beam centers increased again at an altitude of 84 mm. This increase in lateral offset could no longer be corrected above 84 mm, as the vehicle’s translational speed increased, and the resulting inertia outweighed the centering feedback force. Consequently, as shown in Fig. 3c, the vehicle completely deviated from the laser axis at 100 mm. This deviation was consistent with our previous simulations^43–45^, despite the differences between the lightcraft and multi-parabola thruster. This deviation is because in our previous simulations, when an angular offset of more than 3.0 degrees was reached in flight, the centering motion was uncontrolled and led to a deviation from the laser line.

It is assumed that stable beam-riding flight could be achieved without the initial angular offset, as the lateral force induced inside the parabolic mirror would not be generated at altitudes between 40 and 84 mm. As expected from our 6-DOF simulations and experiments^43–46^, this flight stabilization is achieved due to the suppression of blast wave deformation induced in the main parabolic mirror, resulting in feedback only for the angular offset and its reduction. However, this approach to achieving stable flight by adjusting the optical setup will be discussed in a forthcoming paper. Instead of focusing on stable beam-riding flight through optical modifications and eliminating the initial angular offset, we evaluated the criterion for the laser tracking system by measuring the vehicle’s translational velocity (in the *XY*-plane, as indicated in Fig. 3d) before it deviated from the laser axis.

### Criterion of tracking system

Figure 3d shows the time evolution of the vehicle’s velocities in the *X*- and *Y*-directions ($v_x$ and $v_y$ in Fig. 3d, respectively) calculated from the flight trajectory during the free-flight experiment. The translational velocities $v_x$ and $v_y$ were evaluated from the vehicle’s positions every 20 ms, corresponding to the repetition period of the laser used in this experiment. The timing of the pulse irradiation is also shown in Fig. 3d. 12 laser pulses were irradiated onto the vehicle from launch until deviation. The vehicle reached an altitude of 40 mm at 0.125 s and began to deviate from the laser beam line. The flight altitude gradually increased to 100 mm by 0.22 s, after which the vehicle completely deviated from the laser beam. Following this deviation, the translational velocity of the vehicle reached a maximum value of 0.25 m/s at 0.24 s.

To determine the criteria for the actuation speed of the active tracking system, we analyzed the time evolution of the vehicle’s translational velocity. The active control system for maintaining beam-riding flight can be effective between the onset of deviation and before complete deviation from the laser beam line. Assuming the active control system can restore the vehicle’s position and posture to a stable state within three pulses of laser irradiation after deviation begins, Fig. 3d indicates that the maximum vehicle velocities during these three pulses can be 0.08 m/s (the shaded area in Fig. 3d shows three pulses after deviation starts). Therefore, to develop an effective active control system, we set a target actuation speed of more than 0.08 m/s, which is greater than the translational velocity before the vehicle deviates.

Here, the vehicle velocities were measured based on stereo camera imaging; however, the binarization process of the camera image had an error of 0.5–1 pixels for each frame. These binarization errors could induce position detection errors of approximately 0.20 mm in our stereo camera setup. If the typical moving distance of the vehicle is 2.0 mm during a 50-Hz repetition frequency of the pulse, a 10% error could be included in the velocity estimation. From this error estimation, the vehicle speed measured at 0.08 m/s actually had a variation range of 0.072–0.088 m/s due to binarization error. The measurement error of the vehicle speed could be decreased if the accuracy of the binarization process is improved, which could be future work for actual launch missions.

## Results in tracking experiment

### Output voltage measurement of sound sensor for fixed vehicle

**Fig. 4:**
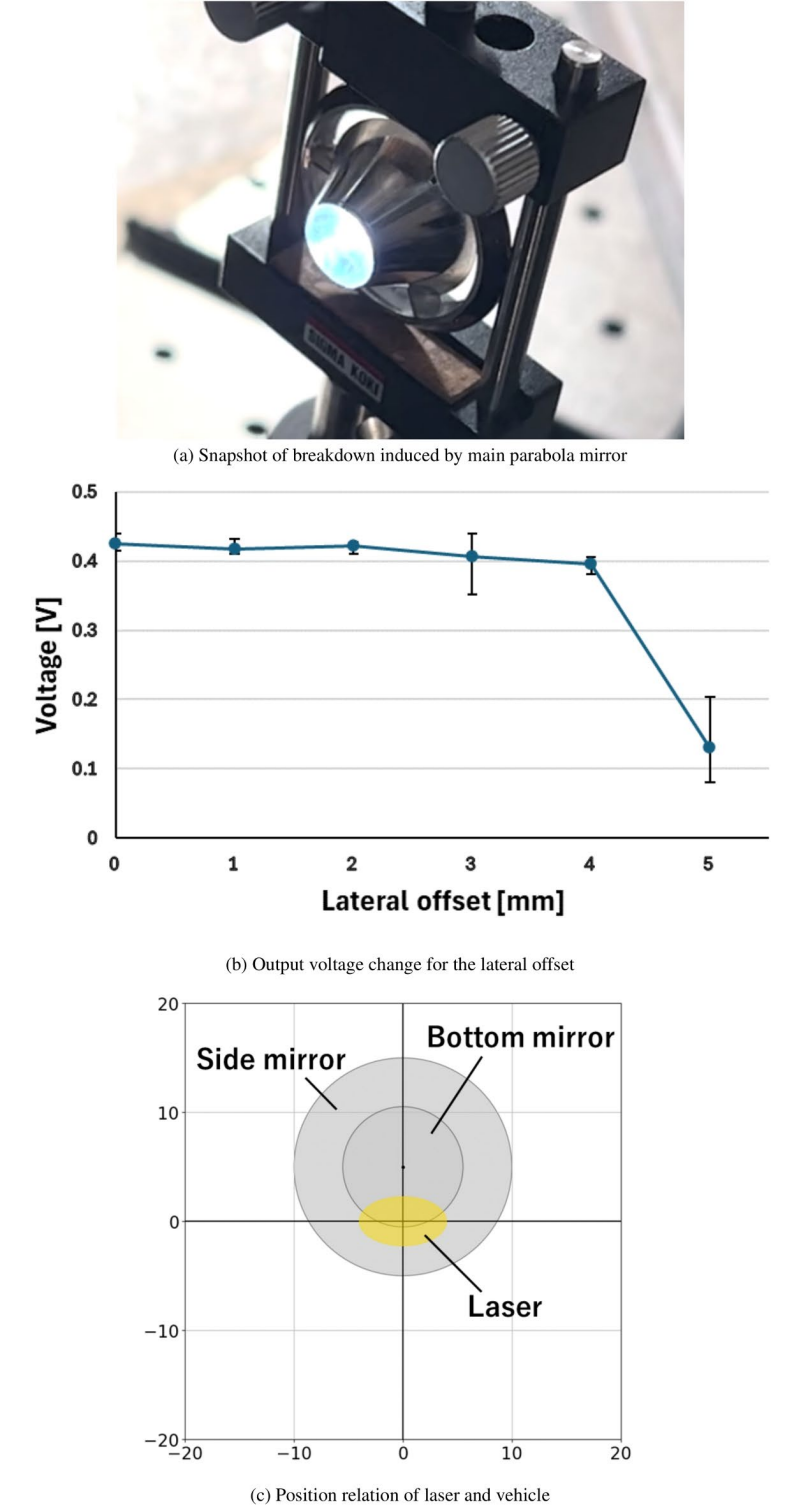
Breakdown experiment to measure output voltage.

To establish the criterion for successful tracking, we measured the change in the output voltage of the sound sensor by irradiating a fixed vehicle with a laser pulse and varying the lateral offset. This was conducted before the tracking experiment for the moving target. Figure 4a shows a snapshot of the breakdown induced by the main parabolic mirror on a multi-parabola vehicle, captured by an iPhone at a resolution of 1, $080 \times 1$,920 pixels and a frame rate of 60 fps. As shown in Fig. 4a, breakdown was successfully achieved, and laser-induced plasma was generated when the 450-mJ laser pulse irradiated the main parabolic mirror. By capturing the explosive sound associated with this laser breakdown, we can verify successful laser irradiation by the tracking system. Furthermore, assuming that the sound level could change depending on the lateral offset between the laser and vehicle axes, we measured the change in the output voltage of the sound sensor by varying the lateral offset while maintaining an angular offset of 0 degrees (Fig. 4b). The sound was measured three times for each laser offset.

Figure 4b reveals that the sound level was high at a lateral offset of 4 mm or less but drastically decreased at a 5-mm lateral offset. This is because the laser irradiation position shifted from the center to the side mirrors as the lateral offset increased. With this shift, the point focus, induced in the center mirror with high laser energy density, changed to a ring-shaped focus with lower energy density, as shown in Fig. 4c. Due to the decrease in energy density at the focal point, the shock wave strength and sound level weakened at a lateral offset of 5 mm. From this sound level measurement, we determined that the laser irradiation position is within 4 mm of the vehicle center if the output voltage exceeds 0.35 V. Consequently, in this experiment, an output voltage greater than 0.35 V will be considered a successful outcome of the tracking process.

### Tracking experiment for moving target

**Fig. 5:**
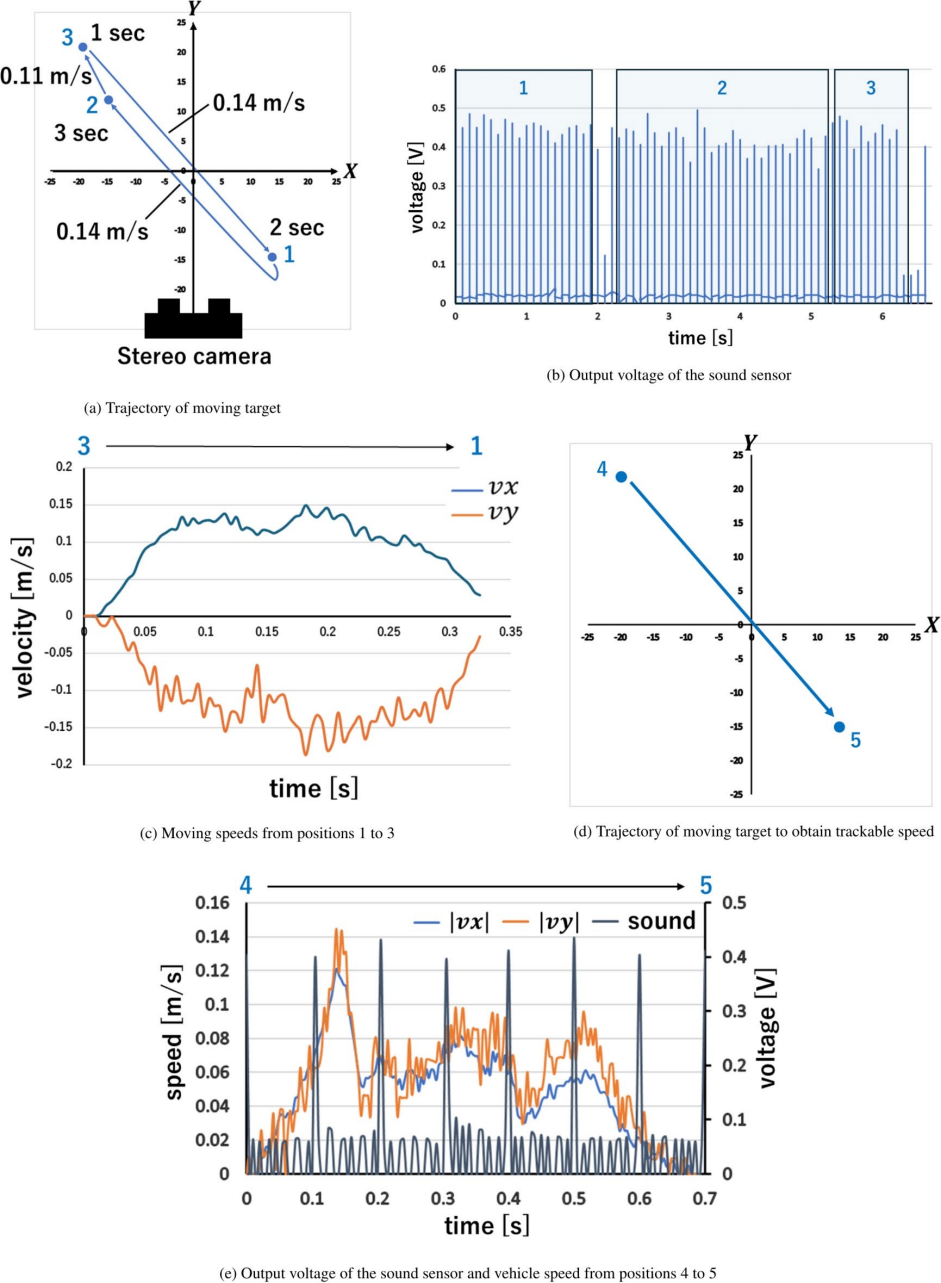
Tracking experiment for a moving target.

Quantitative performance evaluation of the tracking system was not possible due to the high DOF of motions when tracking was conducted for the free-flight vehicle from the beginning. Therefore, a robotic arm system was developed to evaluate the performance of the tracking system by simulating the free-flight motion while restricting the DOF to translational motion only. A tracking experiment was then conducted by moving a vehicle in the *XY*-plane using a robotic arm. Figure 5a shows the vehicle’s trajectory in the *XY*-plane. The vehicle waited for 2 seconds at position 1, moved to position 2 at an average velocity of 0.14 m/s, waited for 3 seconds at position 2, moved to position 3 at an average velocity of 0.11 m/s, waited for 1 second at position 3, and finally returned to position 1 at an average velocity of 0.14 m/s. The time-averaged velocities of the target were calculated by dividing the distance between each position by the time required, which was adjusted to approximately match the maximum velocity expected to be trackable by the laser control system ($v_\textrm{max}=0.126$ m/s, where $v_\textrm{max}$ is the trackable speed estimated in the section of methods). The instantaneous velocity of the vehicle differs from the average velocity due to the transient response of the robot arm motor. Additionally, the oblique motion of the vehicle in the *XY*-plane was chosen to drive and test the two linear guides.

Figure 5b shows the time evolution of the output voltage of the sound sensor during the tracking experiment. The areas enclosed by the light blue squares represent the stationary states of the vehicle, and the areas between the squares represent the moving vehicle states. The numbers in the light blue squares indicate the target positions. Although the output voltage of the sound sensor fluctuated, the laser pulse was successfully irradiated onto the vehicle’s center mirror when the vehicle was stationary, as the output voltage exceeded 0.35 V in these areas. The output voltage fluctuations were likely caused by variations in incident laser energy and errors in image processing; however, there were no significant misses during the stationary state. Some output voltages fell below 0.35 V when the vehicle moved from positions 1 to 2 and positions 3 to 1, indicating tracking failures during these transitions.

The cause of tracking failure was determined to be the vehicle’s moving speed exceeding the maximum trackable speed of $v_\textrm{max}=0.126$ m/s. This is evident when the instantaneous vehicle velocity from positions 3 to 1 over time, as illustrated in Fig. 5c. The instantaneous velocity, calculated from a series of snapshots, showed that the vehicle’s maximum translational speed reached 0.186 m/s, surpassing the maximum trackable speed of $v_\textrm{max}=0.126$ m/s. Since tracking failure is attributed to this overspeed, the next subsection evaluates the trackable speed by gradually increasing the vehicle’s speed.

### Evaluation of trackable speed

To determine the range of moving speeds that allow accurate tracking without irradiation misses, we incrementally increased the vehicle’s speed. In this experiment, the vehicle moved from positions 4 to 5, as shown in Fig. 5d. This oblique movement from positions 4 to 5 was counted as one cycle, and the cycle was repeated. The vehicle’s moving speed was increased for each cycle by controlling the motor’s rotational speed. Finally, we identified the upper limit of the vehicle’s moving speed at which tracking was successful for all laser pulses during a cycle. Successful tracking was confirmed when the output voltage of the sound sensor exceeded 0.35 V for each laser shot during a cycle.

Figure 5e shows the output voltage of the sound sensor and the vehicle’s moving speed when all laser shots were successful during a cycle. $v_x$ and $v_y$ in Fig. 5e represent the *X*- and *Y*-components of the vehicle velocity vector, respectively. The vehicle’s moving velocity exhibited temporal variations due to the unstable rotation speed of the servomotor used in the robot arm. However, an output voltage exceeding 0.35 V indicated successful tracking for each timing of the laser irradiations. From Fig. 5e, the trackable speed of our system can be evaluated as 0.09 m/s, as this was the maximum speed of the vehicle at the time of pulse irradiation during the oblique motion. Although the maximum translational velocity of the vehicle before deviation, as evaluated in the free-flight experiment, was 0.08 m/s, our system’s trackable speed of 0.09 m/s exceeds this, ensuring effective tracking. Although the laser pulse energy used in this tracking experiment was limited to approximately 450 mJ, which is insufficient to levitate a vehicle in the air, we plan to apply our tracking system to a free-flight experiment with a high-power laser source in a future study.

## Conclusion

A free-flight experiment on the multi-parabola thruster was conducted to understand the flight dynamics of a beaming vehicle and establish criteria for the tracking system. Repetitive pulses with a pulse energy of 4.93 J and a repetition frequency of 50 Hz were applied to the vehicle, which was placed on a rotating stage to provide an initial axial spin, thereby stabilizing the beaming flight through the gyro effect. A vehicle with a mass of 2.17 g was launched with an initial axial spin of 7,800 rpm, successfully propelled by the laser-induced blast wave. However, the vehicle deviated from the laser beam line during repetitive pulse irradiation due to nonparallel beam formation and the initial angular offset caused by the laser setup. Consequently, the maximum flight altitude achieved in the free-flight experiment was 110 mm.

In addition to the free-flight experiment, a tracking system was developed to enhance stability during beaming flight. The performance requirements for the tracking system were determined based on detailed free-flight dynamics data. Monitoring the vehicle motion with cameras revealed that the maximum translational speed of the vehicle in the *XY*-plane, excluding the vertical upward direction, was 0.08 m/s just before complete deviation occurred. This indicates that the translational speed of the laser position in the tracking system should exceed 0.08 m/s in the *XY*-plane. Additionally, to meet the time-response requirement, the entire tracking process must be completed within 20 ms to synchronize with the laser system’s repetition frequency.

After establishing the performance requirements, we developed a tracking system by installing laser reflection mirrors on linear guides. These mirrors were driven by high-speed stepping motors and motor drivers to meet the specified performance criteria. In this tracking system, the vehicle’s motion was captured using a stereo camera system, which determined the laser position for the next pulse irradiation. The entire image processing was completed within 6–10 ms, shorter than the 20-ms repetitive pulse period.

For the tracking experiment, a robot arm was developed to move the target (the multi-parabola thruster) in the *XY*-plane. The target was mounted on the robot arm, and *XY*-plane motion was applied while maintaining an angular offset of zero degrees. For safety reasons, 450-mJ repetitive pulses were used instead of the 4.93-J pulses from the free-flight experiment. Although the laser energy was lower, it was sufficient to induce breakdown when the vehicle’s main parabolic mirror was successfully irradiated, with breakdown sounds confirming successful tracking. The system’s trackable speed was evaluated by gradually increasing the target’s moving speed and detecting breakdown sounds using a sound sensor. This experiment showed that the maximum trackable speed in the *XY*-plane was 0.09 m/s, exceeding the tracking system requirement of 0.08 m/s estimated from the free-flight experiment. In future studies, the proposed tracking system will be applied to free-flight experiments conducted at a higher-averaged-power laser facility.

Additionally, the experiments described in this paper have been conducted under ideal conditions; however, practical launch situations include uncertain disturbances arising from long-distance laser beam propagation (until 100 km), wind, and laser tracking errors. Robust flight control based on the tracking system should be achieved to maintain stable flight under these disturbances and uncertainties. Our previous 6-DOF simulations with perturbations indicated that robust flight with beam-riding is possible using the tracking system if the laser irradiation position is suitably selected based on an optimization algorithm^43–45^; therefore, the flight experiments for such robust control will be discussed in a future work.

In addition to wind and laser tracking errors, beam diffraction and beam distortion caused by atmospheric disturbances can cause an issue for our flight control technology. Therefore, a discussion on these topics is crucial. The beam diameter may increase due to the diffraction effect during long-distance transmission, such as over 100 km in air; however, this divergence effect can be mitigated by selecting a beam source with a shorter wavelength and wider waist. For example, if a Gaussian beam with a beam waist of 0.5 m and wavelength of 10.6 $\mathrm{\mu m}$ is used as the energy source, the beam diameter at 100-km altitude can be evaluated to be 1.5 m. Additionally, the vehicle diameter in actual launch missions will be enlarged compared to that of the experimental model, which can be 2–10 m to transport a payload satellite or materials. In this case, because the beam diameter remains within the diameter of the vehicle’s body and ring-shaped cowl even at a 100-km altitude, controlling the beam irradiation position can sufficiently effect the thruster’s impulse direction control. In addition, the beam pattern, diameter, and power are affected by fluctuation in atmospheric density and temperature during long-distance transmission, as discussed in previous work^47^, which substantially impact flight control accuracy when using laser position control. The beam fluctuation can also be enhanced by dust and plasma effects in the ionosphere. Given that these perturbation effect on the beam transmission cannot be overlooked, an adaptive optics approach will be required in actual launches to compensate for laser propagation from the Earth’s surface to high altitudes. Previous work^48^ indicated that a combination of downlink laser from the vehicle and deformable mirror is effective for compensating for the laser beam propagation from the ground to space. Additionally, the performance of the adaptive optics system was experimentally examined using an actuator mirror, demonstrating that real-time correction for the beam propagation is possible^49^. Because adaptive optics approaches may also be effective for our flight system, the adaptation of these systems for actual launch missions involving high-power repetitive pulse lasers will be discussed in future work.

As future applications to actual launch missions of laser propulsion vehicles, the stereo camera in the tracking system should be replaced with a telescopic camera to monitor the vehicle in altitudes below 100 km. Additionally, a GPS and multi-axis accelerometer sensor system, as onboard monitoring systems of the flying vehicle, could be combined with the camera monitoring system to improve the flight detection accuracy. In addition, the ground-based mirror actuation system should be enlarged with an increase in incident laser power and flight altitude; however, enlargement using a single actuation device has a size limitation due to the mirror’s moment of inertia and actuator torque. In this case, the high power laser is divided into several lines and its irradiation positions on the vehicle are actively changed through several small actuators to suppress the increase in mirror inertia mounted on each actuator. Finally, each laser path could be bundled and irradiated onto the flying vehicle while maintaining high power density of the laser, which could propel the vehicle with a mass of more than 10 kg, as discussed in our previous scaling study^45^.

## Methods of free-flight experiment

To understand the performance requirements of the tracking system, a free-flight experiment was conducted using $\hbox {CO}_2$ repetitive pulses and a multi-parabola thruster.

### Multi-parabola thruster

In this experiment, we used the multi-parabola thruster instead of a lightcraft. Although the development and performance evaluation of the multi-parabola thruster are not the main focus of this study, and thus detailed design processes using computational fluid dynamics simulation and 6-DOF impulse measurement are not presented here, we provide an overview of the multi-parabola thruster.

**Fig. 6:**
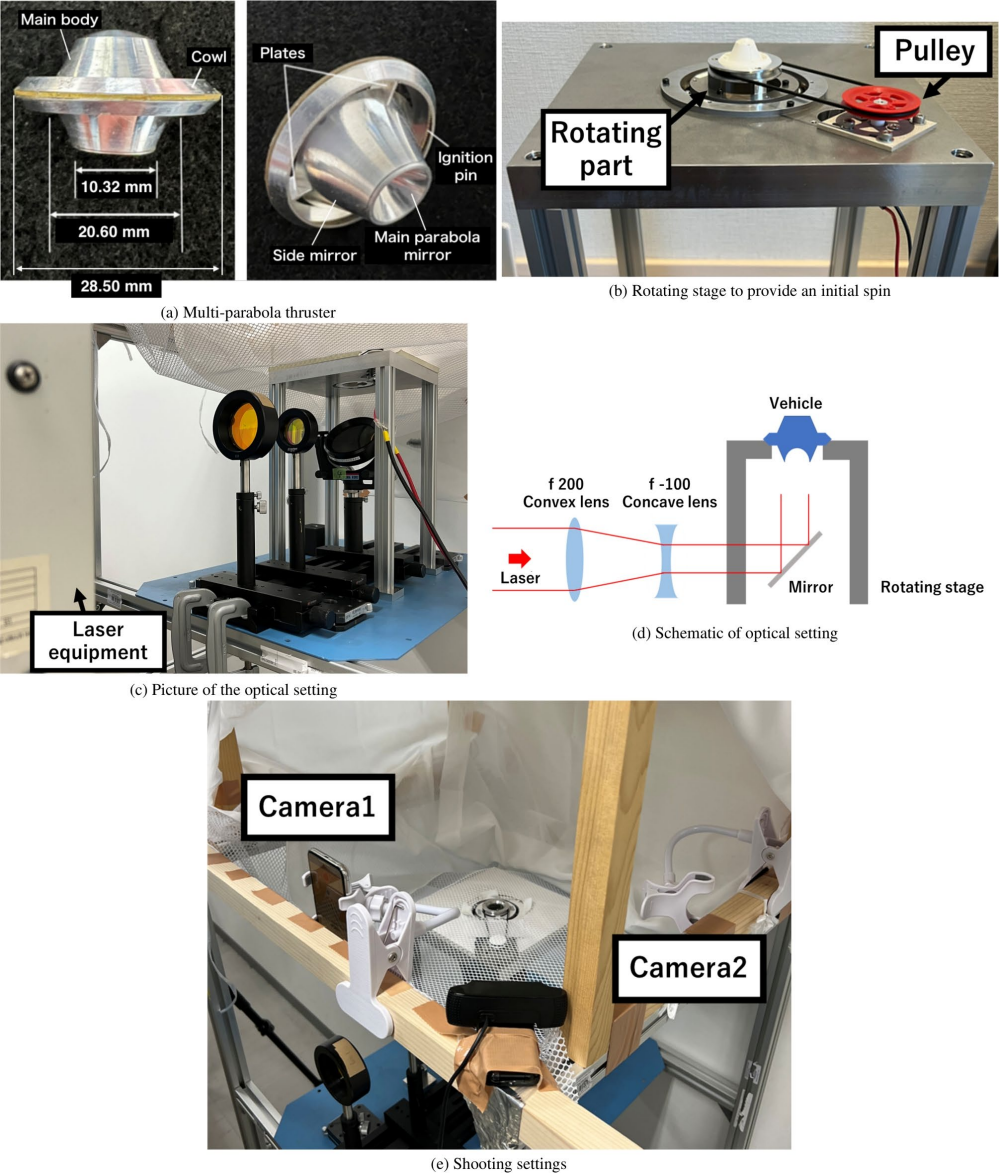
Experimental setting for free flight.

The multi-parabola thruster was developed by combining the lightcraft proposed by Myrabo^26–28^ and the simple parabola thruster proposed by DLR^29–31^. It features a ring-shaped cowl, a simple parabolic mirror, and a spike-nozzle-shaped side mirror (Fig. 6a). Axial thrust is generated by the simple parabolic mirror when the laser pulse irradiates the vehicle because a spherical blast wave is generated from the parabola’s focal point. Additionally, similar to the simple parabolic thruster proposed by DLR^29–31^, an angular restoring impulse is generated by the parabolic mirror when there is an angular offset between the laser and vehicle axes. This occurs because the focal point shifts due to the angular offset, altering the blast wave’s generation position. Moreover, the side mirror, akin to the one used in the lightcraft, can create a ring-shaped blast wave that interacts with the cowl. This annular blast wave pushes the vehicle back to the center of the beam when a lateral offset between the vehicle and the incident beam axes occurs.

As shown in Fig. 6a, the multi-parabola thruster used in this free-flight experiment featured a ring-shaped cowl with a diameter of 28.50 mm, a main parabolic mirror with a diameter of 10.32 mm, and a side mirror with a diameter of 20.60 mm. The focal length of the main parabolic mirror is 1.0 mm. Super duralumin was used to minimize the total vehicle mass. The weight of the multi-parabola thruster was set at 2.17 g, and the moments of inertia for roll, pitch, and yaw were 195.81, 126.58, and 126.58 g$\cdot \mathrm {mm^2}$, respectively.

### Rotating stage to provide an initial spin

The multi-parabola thruster was designed to provide greater flight stability than the lightcraft-type vehicle. However, applying initial spin around the vehicle axis can further enhance flight stability due to the gyro effect. To achieve this, a rotating stage was developed to import initial rotation to the multi-parabola thruster (Fig. 6b). This stage comprised a deep-groove ball bearing (6010, NTN), a torque tune motor (370 motor, TAMIYA), a pulley, a rubber belt, and a DC-stabilized power supply (PSW-360L30, TEXIO Technology Corporation). The DC power supply was connected to the motor, rotating an 18-mm diameter pulley mounted on the motor. This rotation was transmitted to the bearing via a rubber belt connected to a rotational transmission ring with a diameter of 35 mm. The bearing’s maximum rotational speed was 7,800 rpm, and the vehicle was mounted on the rotating bearing to provide initial rotation before laser pulse application.

### Optical and shooting settings

A repetitive $\hbox {CO}_2$ laser beam (ML205E, SLCR-Lasertechnik GmbH) was used for free-flight experiments. The wavelength of the laser was 10.6 $\mathrm{\mu m}$, and the maximum repetition frequency was 50 Hz. The full width at half maximum of the primary laser power was 0.2 $\mathrm{\mu s}$. After the power peak, 90% of the total beam energy was emitted within 3 $\mathrm{\mu s}$. A repetition frequency of 50 Hz was chosen to provide sufficient thrust to the multi-parabola thruster mounted on the rotating stage. The beam pattern emitted from the injection port was elliptical with long and short diameters of 47 mm and 34 mm, respectively. To increase the laser fluence applied to the vehicle and generate a stronger blast wave, the beam radius was reduced using a ZnSe plano-convex lens with a focal length of 200 mm (SIGMAKOKI) (Fig. 6c,d). Collimation of the focused beam was then achieved using a ZnSe plano-concave lens with a focal length of −100 mm (SIGMAKOKI), resulting in an elliptical beam with long and short diameters of 11 mm and 10 mm, respectively. Typically, the distance between lenses should be 100 mm to produce parallel beams. However, to achieve higher laser energy density, the distance between lenses was set to 125 mm, reducing the beam diameter further and resulting in a nonparallel beam formation. The effects of this nonparallel beam formation are discussed below. The beam patterns measured in the experiment are also described in the following sections. The laser’s incident angle was adjusted from horizontal to vertical using a flat mirror, and the laser was irradiated onto a vehicle mounted on the rotating stage. Using a power meter (ED-500IR, Gentec-EO) and a digital oscilloscope (DSC-1074B, TEXIO), the laser energy reflected from the flat mirror was measured to be 4.93 J.

The flight motion during repetitive pulse irradiation was recorded using two cameras (iPhones with a frame rate of 240 fps in slow-motion mode and 12 million pixels, Apple). The cameras were mounted on the frame surrounding the vehicle and rotating stage, arranged perpendicularly to each other (Fig. 6e). A blackout curtain was placed around the frame during the flight experiment to protect us from unexpected laser reflections.

## Methods of tracking experiment

### Real-time target detection in the three-dimensional space

**Fig. 7:**
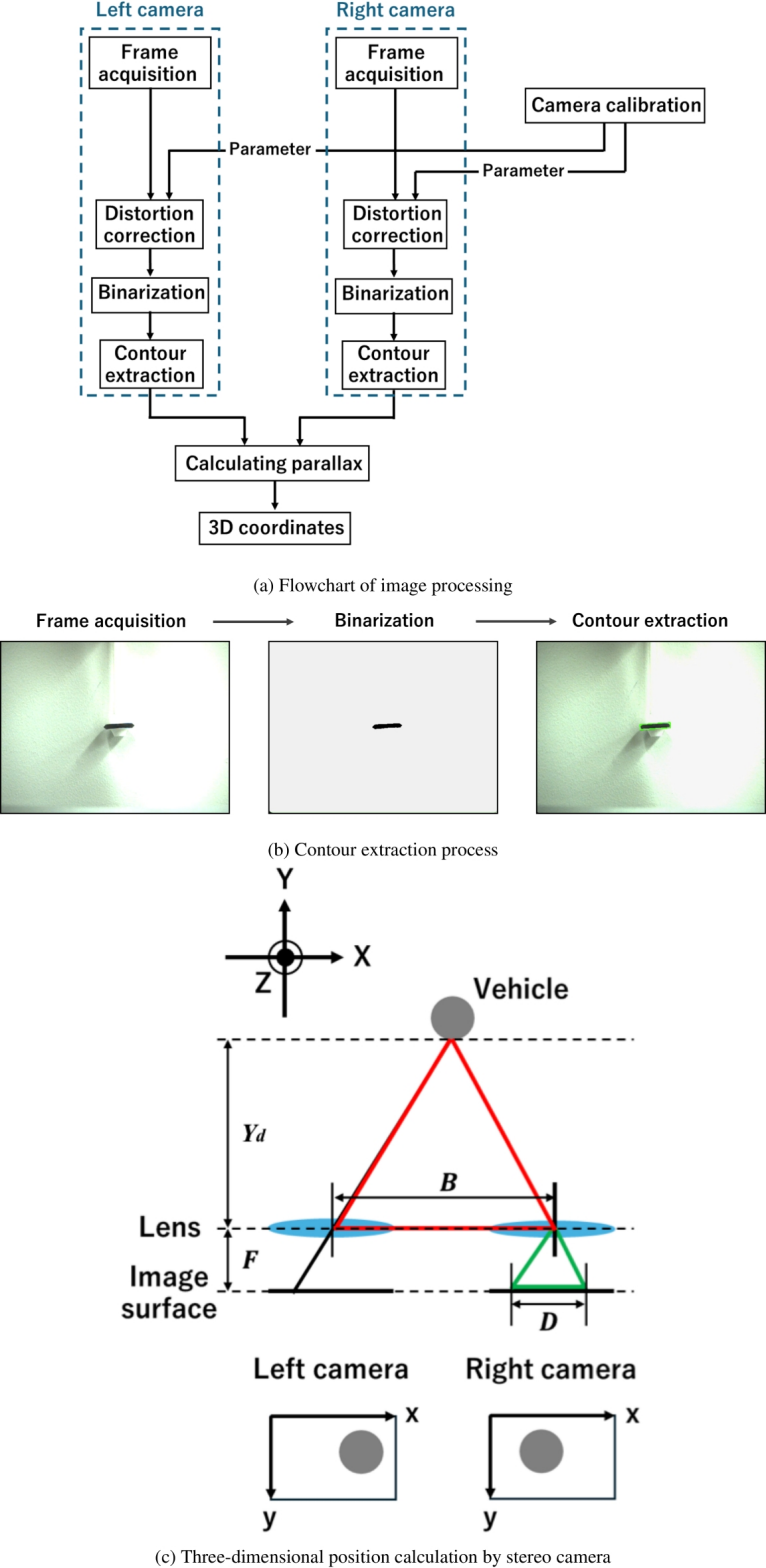
Flowchart of image processing.

The real-time target detection system was developed by connecting two USB cameras (STC-MCS43U3V, 450 fps, $720 \times 540$ pixels, OMRON Corporation) to a PC (Mac mini 2020, eight cores, 16 GB memory, Apple) and using them as stereo cameras. The vehicle’s translational position in three-dimensional (3D) *XYZ*-space was calculated in real time based on images captured by the stereo cameras. This study presents real-time 3D position calculations, but our method can be upgraded to calculate the vehicle’s attitude and angular velocity in addition to translational motion. Real-time monitoring of the vehicle’s attitude and angular velocity will be discussed in a future study. Figure 7a shows a flowchart of the video shooting, image processing, and calculation of the 3D translational position of the vehicle.

Before calculating the 3D position, the two-dimensional (2D) positions of the vehicle were evaluated from each frame image captured by the two cameras. This was achieved through frame acquisition, image binarization, and contour extraction, as shown in Fig. 7b. The 2D information was then reconstructed into 3D information. During the 2D information detection process, image binarization was conducted using OpenCV to isolate the vehicle. To facilitate this binarization process, the cowl of the vehicle was colored black, and the background was white to help separate the vehicle from the background. After obtaining the binarized image, contour extraction was performed by checking the luminance of each pixel using the OpenCV function. The position of the center of gravity in the 2D plane was then determined in real time by fitting the external minimum rectangle to the vehicle’s cowl.

Each 2D position captured by the two cameras was converted to 3D coordinates through geometric calculations, as shown in Fig. 7c. If the *x*- and *y*-coordinates are defined in the images captured by each camera, and each camera is set horizontally (Fig. 7c), the *x*-position of the vehicle can vary while maintaining the same *y*-position. This *x*-position difference is called parallax (*D*), which can be used to estimate the depth $Y_d$ based on the scalene triangle (red and green triangles in Fig. 7c) using the following formula:

1 $$\begin{aligned} Y_d=\frac{B \cdot F}{D}, \end{aligned}$$

where *B* is the distance between each camera, and *F* is the focal length of the lens. By applying this equation, the 3D position of the vehicle was obtained using a stereo camera. In our experiment, the distance between the cameras (*B*) was 10 cm, and the focal length of the lens (FA0401C, Chiopt Corporation) (*F*) was 4 mm.

Camera calibration is also necessary to improve image processing accuracy, as individual cameras and lenses have unique characteristics. Calibration was performed by photographing a known flat pattern (a chessboard pattern was used in this experiment) and comparing the captured image with the known coordinates of the pattern. As a result, the camera matrices for the left and right cameras, used to convert camera coordinates to image coordinates, were obtained as follows:

2 $$\begin{aligned} \text {Camera matrix (left)}= \begin{bmatrix} 607.1890 & 0 & 365.1721 \\ 0 & 607.0821 & 281.1172 \\ 0 & 0 & 1 \end{bmatrix}, \end{aligned}$$

3 $$\begin{aligned} \text {Camera matrix (right)}= \begin{bmatrix} 604.9491 & 0 & 356.1752 \\ 0 & 605.4161 & 270.3537 \\ 0 & 0 & 1 \end{bmatrix}. \end{aligned}$$

Additionally, the distortion coefficients of the lenses, which are correction coefficients to suppress distortion aberration, were determined:

4 $$\begin{aligned} \text {Distortion coefficients (left)}= \begin{bmatrix} -0.0926&0.1024&0.0002&-0.0007&-0.0613 \end{bmatrix},\end{aligned}$$

5 $$\begin{aligned} \text {Distortion coefficients (right)}= \begin{bmatrix} -0.0968&0.1051&0.0000&-0.0010&-0.0240 \end{bmatrix}. \end{aligned}$$

These matrices and coefficients were applied to the pixel position vector to correct frame image distortion, as shown in Fig. 7a. The entire image processing sequence shown in Fig. 7a was completed within 6–10 ms, which is less than half the pulse period (20 ms) of the laser beam. Controlling the beam irradiation position begins with this image processing.

### Control of beam irradiation position

**Fig. 8:**
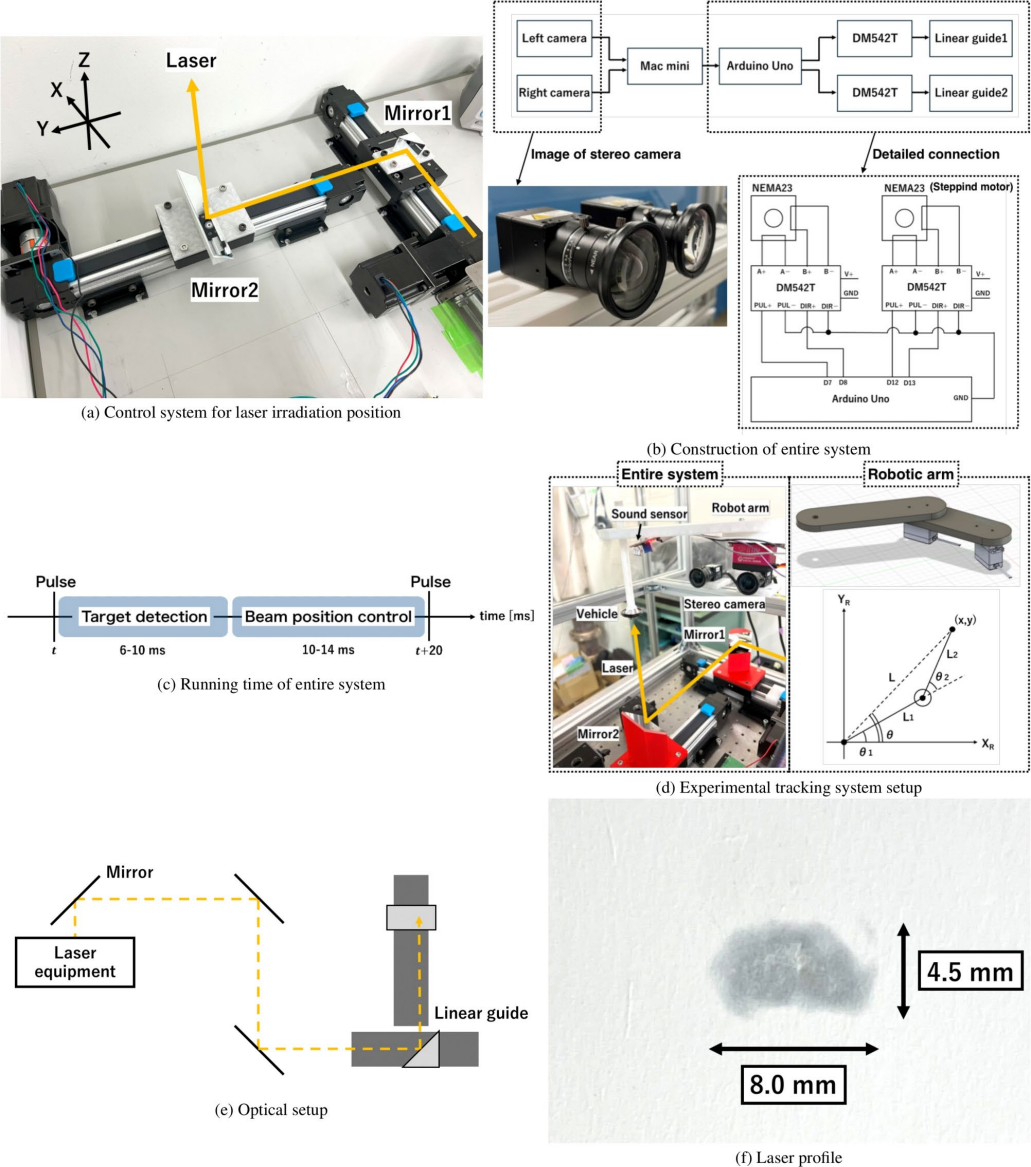
Complete system for image processing and laser position control. Setup for the tracking experiment.

We developed a method to control the laser irradiation position based on the vehicle’s 3D position information obtained through image processing while maintaining the laser incident angle perpendicular to the ground. Two flat mirrors were mounted on linear guides (Heechoo Corporation) driven by stepping motors (Oukeda NEMA 23 Stepping Motor OK57STH56-2804AD8, OUKEDA MOTOR) and stepping motor drivers (DM542T, Leadshine) to establish a control system for the laser irradiation position, as shown in Fig. 8a. The Arduino UNO controls the two DM542T motor drivers after receiving the vehicle position information from the PC. The entire system, including image processing and laser position control, is illustrated in Fig. 8b. The incident laser beam was reflected by mirrors 1 and 2, altering the propagation angle towards the vertical direction. The laser irradiation position was controlled in the *XY*-plane by driving the stepping motors and adjusting the positions of the two mirrors. The step angle of the motor was 0.45 degrees, resulting in a moving resolution of 0.09 mm for the mirrors. The step angle error of the motor was approximately 5%, which could induce a 5% error in mirror positioning. However, this positioning error was sufficiently smaller than the target diameter when the typical displacement of the mirror during a 50-Hz repetition frequency of the laser was in the range of 1–10 mm. Due to small actuation errors, similarities between each experiment could be maintained.

Based on the rotational speed of the stepping motor, the maximum moving speed of the mirrors ($v_\textrm{mr}$) was expected to be 0.18 m/s in our developed tracking system. However, it is important to note that this moving speed is not the allowable target moving speed for laser irradiation at a 50-Hz repetition frequency (the interval between each laser pulse is 20 ms). Since the target detection process takes 6–10 ms before beam position control, the available time for position control ($T_\textrm{op}$) is limited to 10–14 ms to complete the entire operation before the next pulse, as shown in Fig. 8c. Assuming the vehicle moves at a speed of *v* m/s during the 20-ms pulse period, the vehicle’s displacement would be 0.02*v* m at the next pulse timing. Therefore, to ensure accurate tracking, the mirrors must move a distance of 0.02*v* m within the operation time $T_\textrm{op}$ of 10–14 ms. This means that the maximum velocity that can be tracked by the laser control system ($v_\textrm{max}$) is evaluated using the formula: $0.02 v_\textrm{max}=v_\textrm{mr} \cdot T_\textrm{op}$ m/s. The value of $v_\textrm{max}$ can be calculated in a range from 0.09 to 0.126 m/s, based on $v_\textrm{mr}=0.18$ m/s and $T_\textrm{op}=$10–14 ms. This estimated speed exceeds the goal vehicle speed of 0.08 m/s estimated from the free-flight experiment discussed above, indicating that the developed system can be effective for flight control.

### Setup of tracking experiment

Figure 8d shows the experimental setup of the tracking system. To prepare for the tracking system’s adaptation to the free-flight experiment, the vehicle was moved in the translational *XY*-direction (while its height was fixed) by mounting it on a robot arm. Although the angular offset between the laser and vehicle axes could affect the flight stability, in this study, for simplicity, the laser and vehicle axes were kept parallel by controlling the mirrors mounted on the laser-tracking system in the *XY*-plane. The effect of the angular offset during the tracking experiment will be evaluated in a forthcoming paper. The robot arm was fabricated using a 3D printer, and its motion was controlled using servomotors (DS3218, DSSERVO Inc.) and an Arduino UNO. The detailed design of the robot arm is shown in the right side of Fig. 8d. The arm lengths $L_1$ and $L_2$ were 100 and 150 mm, respectively. The motor rotation angles $\theta _1$ and $\theta _2$ were determined to move the target position to (*x*,  *y*) in the robotic arm coordinate system of $X_RY_R$ as follows:

6 $$\begin{aligned} \theta _1= \theta -\textrm{cos}^{-1}\frac{L^2+L_1^2-L_2^2}{2LL_1}, \end{aligned}$$

7 $$\begin{aligned} \theta _2=\pi -\textrm{cos}^{-1}\frac{L_1^2+L_2^2-L^2}{2L_1L_2}, \end{aligned}$$

where $\theta =\textrm{tan}^{-1}(y/x)$ and $L=\sqrt{x^2+y^2}$. The speeds of these rotations were controlled based on the VarSpeedServo library. The multi-parabola vehicle, which had a cowl with a diameter of 28.20 mm, a side mirror with a diameter of 20.00 mm, and a main parabola mirror with a diameter of 11.04 mm, was mounted on the tip of the robot arm. This vehicle served as the target for the tracking system, with the laser irradiation target set at the vehicle’s center of gravity. For this moving target, a series of position detections, mirror adjustments, and laser irradiation processes were conducted using our tracking system and a repetitive laser pulse. To detect the vehicle motion, a stereo camera system was mounted on a frame. The surface of the vehicle’s cowl was coated with black paint to improve the detection accuracy of the tracking system.

Repetitive laser pulses were generated from a YAG laser source (Nd:YAG LASER SYSTEM LS-2137/2, LOTIS TII Inc.) and directed towards mirrors equipped on the actuation system. The laser had a wavelength of 1,064 nm, and a repetition frequency of 10 Hz. The laser pulse output from the source was reflected by three silver-coated flat mirrors (ME1-P01, Thorlabs Inc.) and introduced into the linear guide, as shown in Fig. 8e. The laser’s propagation direction was then changed to vertical upward through reflections using two silver-coated flat mirrors (ME1-P02, Thorlabs Inc.) mounted on the linear guide. Given the mirror size on the linear guide was 50.$8 \times 50$.8 mm, the trackable range in this experiment was also 50.$8 \times 50$.8 mm, which was deemed sufficient for free-flight experiments. Figure 8f shows the laser pattern captured immediately before the laser irradiated the vehicle, displaying an elliptical pattern with major and minor axes of 8.0 mm and 4.5 mm, respectively. Laser energy was measured using a power meter (ED-500 LIR+, Gentec-EO) and a digital oscilloscope (MSO5074, RIGOL), registering approximately 450 mJ immediately before the laser irradiated the vehicle. For safety reasons, the laser energy in the tracking experiment was lower than in the free-flight experiment; however, it was sufficient to induce gas breakdown when the vehicle’s parabolic mirror received a laser pulse.

The generation of an explosive sound indicated the successful completion of the laser breakdown process. To verify the effectiveness of vehicle motion tracking, this breakdown sound was confirmed using a microphone sensor (KY-037, KKHMF) and a digital oscilloscope (MSO5074, RIGOL) for each shot of repetitive pulses. Before conducting the tracking experiment with repetitive pulses, the output voltage of the sound sensor was measured by irradiating a fixed vehicle with a laser pulse to induce a gas breakdown. This output voltage served as the criterion for successful tracking.

## Data Availability

Requests for materials or codes should be addressed to Masayuki Takahashi.
